# Continuation of Selective Alpha Blocker After Transurethral Resection of the Prostate Is Associated with a Decreased Risk of Hip Fractures in Elderly Patients Diagnosed with Benign Prostate Hyperplasia

**DOI:** 10.3390/life15040641

**Published:** 2025-04-13

**Authors:** Wei-Hung Wang, Yi-Ting Hung, Chi Luo, Wen-Tien Wu, Ru-Ping Lee, Ting-Kuo Yao, Cheng-Huan Peng, Hao-Wen Chen, Jen-Hung Wang, Kuang-Ting Yeh

**Affiliations:** 1 School of Medicine, Tzu Chi University, Hualien 970374, Taiwan; 107311149@gms.tcu.edu.tw (W.-H.W.); timwu@tzuchi.com.tw (W.-T.W.); peng0913@tzuchi.com.tw (C.-H.P.); 2 Department of Orthopedics, Hualien Tzu Chi Hospital, Buddhist Tzu Chi Medical Foundation, Hualien 970473, Taiwan; luo91220@tzuchi.com.tw (C.L.); tayao0318@tzuchi.com.tw (T.-K.Y.); charliechen728@tzuchi.com.tw (H.-W.C.); 3 Institute of Medical Sciences, Tzu Chi University, Hualien 970374, Taiwan; fish@gms.tcu.edu.tw; 4 Department of Medical Research, Hualien Tzu Chi Hospital, Buddhist Tzu Chi Medical Foundation, Hualien 970473, Taiwan; paulwang@tzuchi.com.tw; 5 Graduate Institute of Clinical Pharmacy, Tzu Chi University, Hualien 970374, Taiwan

**Keywords:** transurethral resection, hip fractures, benign prostate hyperplasia

## Abstract

Hip fractures significantly affect mortality and quality of life in the elderly population. Although alpha-blockers are commonly prescribed for lower urinary tract symptoms after transurethral resection of the prostate (TURP), their long-term safety regarding fracture risk remains controversial. This study aimed to investigate whether long-term alpha-blocker use after TURP affects the risk of hip fractures requiring surgery in elderly men. This study included 6853 male patients aged ≥50 years who underwent TURP between 2000 and 2018. The alpha-blocker group (*n* = 1371) included patients who continued alpha-blocker treatment after TURP, while the control group (*n* = 5482) included those who had discontinued the medication. The primary outcome was hip fracture requiring surgical intervention. During follow-up (3.80 ± 1.64 years), hip fracture occurred in 4.2% of the alpha-blocker group versus 5.6% of controls. After adjusting for baseline characteristics and competing risk analysis, alpha-blocker use was associated with a significantly lower risk of hip fracture (*p* = 0.005). Subgroup analysis revealed particularly strong protective effects in patients with diabetes. Long-term use of alpha-blockers after TURP was associated with reduced hip fracture risk, particularly in patients with diabetes. These findings suggest the safety of continued alpha-blocker therapy after TURP in these patients.

## 1. Introduction

Hip fractures are a major public health concern, particularly among the elderly population, because they significantly affect mortality, autonomy in daily activities, and the likelihood of requiring institutional care [1]. The 1-year mortality rates following hip fractures are documented to be three to four times higher than those observed in the age-matched general population [2].

Benign prostatic hyperplasia (BPH) is a common age-related condition affecting men, serving as a predominant cause of lower urinary tract symptoms (LUTSs) [3]. The prevalence of BPH demonstrates a clear age-dependent progression, with autopsy studies revealing rates of approximately 8% during the fourth decade of life, increasing to 50% in the sixth decade, and reaching 80% by the ninth decade [4,5]. The progressive nature of BPH and its associated symptoms necessitate long-term management strategies.

In the therapeutic landscape of BPH, alpha-blockers are recommended as the first-line pharmacological treatment for patients presenting with LUTSs [6]. These medications effectively alleviate symptoms by relaxing the smooth muscles in the prostate and bladder neck. However, when conservative medical management is insufficient for addressing LUTSs, surgical intervention, such as transurethral resection of the prostate (TURP), may become necessary. Several population-based studies have indicated an association between alpha-blocker use and an increased risk of falls and fractures among men with BPH [7,8,9,10,11,12,13,14], potentially owing to medication-induced side effects, including dizziness and hypotension [15].

However, these studies primarily focused on short-term outcomes immediately after initiating alpha-blocker treatment, neglecting the long-term implications. This aspect is particularly critical because BPH progresses gradually, often resulting in symptoms that persist for many years, even after undergoing TURP [16]. This is crucial, as post-TURP LUTSs can affect up to 35% of patients [17], while the rates of the use of alpha-blockers post-TURP range from 27 to 46.8% [18,19,20]. Moreover, previous studies have linked both LUTSs [17,18] and alpha-blocker [7,8,9,10,11,12,13,14] usage to an elevated risk of falls and fractures; however, the limited availability of studies poses challenges for physicians in accurately weighing the risks and benefits of managing this medical dilemma.

Therefore, this study aimed to investigate the association between continued alpha-blocker use after TURP and the risk of hip fracture requiring surgery in elderly men, with particular attention to long-term outcomes and potential variations in risk over time. Understanding this relationship is crucial to optimizing post-TURP management strategies and minimizing adverse outcomes in this vulnerable population.

## 2. Materials and Methods

This population-based retrospective cohort study used data from the Taiwan National Health Insurance Research Database (NHIRD). The NHIRD contains comprehensive health and medical treatment data of insured individuals receiving inpatient and outpatient medical care, medication, and surgical treatment. The National Health Insurance (NHI) program provides coverage for >99% of the Taiwanese population, with 97% of Taiwan’s hospitals and clinics registered with the NHI administration. For this study, we randomly selected 2 million beneficiaries with medical records between 2001 and 2017 from among the 23.8 million NHI beneficiaries available in the NHIRD. The age and sex distributions of the sample matched those of the original database population. To ensure privacy, the participant identification numbers were encrypted before data extraction.

This study was approved by the Research Ethics Committee of the Hualien Tzu Chi Hospital, Buddhist Tzu Chi Medical Foundation (IRB 108-242-C). The requirement for written informed consent was waived by the Institutional Review Board. All procedures were performed in accordance with the relevant guidelines and regulations.

The study population comprised male patients aged ≥65 years who underwent TURP for BPH. Patients were categorized into two cohorts: the alpha-blocker cohort, comprising patients who continued alpha-blocker therapy for at least 3 months post-TURP, and the control cohort, comprising patients who did not receive alpha-blocker therapy post-TURP. Patients who had experienced a hip fracture before the index date or had incomplete medical records or follow-up data were excluded. Alpha-blocker continuation was defined as having prescription records for any alpha-blocker medication (tamsulosin, alfuzosin, doxazosin, terazosin, or silodosin) covering at least 80% of days (medication possession ratio ≥ 0.8) during the first 3 months following TURP discharge. This study allowed for gaps between prescriptions of up to 30 days to account for potential delays in prescription refills or follow-up appointments. Patients who received prescriptions covering less than 80% of days during this period or who had no alpha-blocker prescriptions were classified as the control cohort. This definition aligns with pharmacoepidemiologic standards for defining medication adherence in retrospective database studies and provides a reasonable assurance of regular medication exposure during the critical post-surgical period.

To ensure comparability between the cohorts, we performed propensity score matching (PSM) at a 1:4 ratio using a nearest-neighbor algorithm without replacement. The use of 1:4 PSM is intended to enhance statistical efficiency, reduce variability, improve group balance, and maximize data utilization while avoiding the issues associated with overmatching. Propensity scores were calculated using a logistic regression model that included age, sex, index year, and comorbidities. Comorbidities, based on ICD-9-CM and ICD-10 codes, included diabetes mellitus, hypertension, coronary artery disease, hyperlipidemia, chronic kidney disease, chronic liver disease, osteoporosis, and stroke.

The primary outcome measure was defined as any new diagnosis of hip fracture that required surgery (surgery codes 64029 B and 64170 B) after the index date. Follow-up was continued until the date of hip fracture diagnosis, death (defined as withdrawal from the NHI program), or 31 December 2017, which marked the end of the study period.

All statistical analyses were conducted using SAS version 9.4 and Stata version 16 (SAS Institute, Cary, NC, USA). Continuous variables are presented as means and standard deviations, whereas categorical variables are expressed as the number of cases and percentages. For between-group comparisons, continuous variables were analyzed using the Student’s *t*-test, and categorical variables were assessed using either the chi-square or Fisher’s exact test. Statistical significance was set at *p* < 0.05. The association between alpha-blocker use and hip fractures was explored using Cox proportional hazards models, and a multivariate Cox proportional hazards model was employed to calculate adjusted hazard ratios (HRs). Since our target population consists of elderly individuals, we conducted a competing risk analysis to accurately estimate the marginal probability of hip fracture while accounting for mortality as a competing event. A cumulative incidence function plot was made for displaying the cumulative incidence of hip fractures over the follow-up period for both the alpha-blocker and the control cohorts, while accounting for death as a competing risk.

## 3. Results

### 3.1. Baseline Characteristics

After conducting a 1:4 PSM procedure, we analyzed data from 6853 patients, including 1371 in the alpha-blocker cohort and 5482 in the control cohort. Demographic characteristics were well balanced between the two groups (Table 1). The mean age was comparable between the cohorts (alpha-blocker: 72.24 ± 7.69 years; control: 72.26 ± 7.58 years; *p* = 0.906). The distribution of comorbidities showed no significant differences between the two groups (Table 1), with hypertension being the most prevalent (48.4%), followed by diabetes mellitus (21.2%), coronary artery disease (16.4%), hyperlipidemia (14.3%), and cerebrovascular accidents (13.3%). The less common comorbidities included chronic liver disease (4.5%), osteoporosis (1.5%), and chronic renal failure (1.4%). This balanced distribution of baseline characteristics demonstrated the effectiveness of our PSM approach in minimizing potential confounding factors.

### 3.2. Primary Outcome Analysis

**Figure A1:**
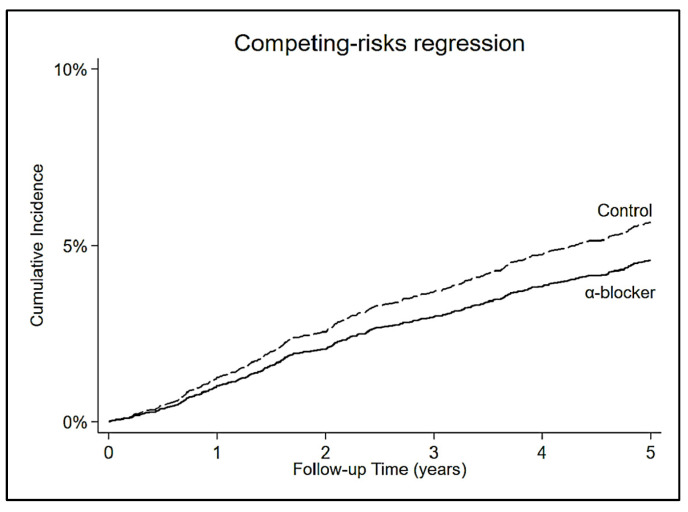
The cumulative incidence of hip fractures in the alpha-blocker cohort and in the control cohort.

In the analysis of hip fracture incidence (Table 2), we observed 83 hip fractures in the alpha-blocker cohort and 541 hip fractures in the control cohort, corresponding to incidence rates of 11.6 and 13.7 per 1000 person-years, respectively. The initial analysis showed a trend toward reduced risk in the alpha-blocker cohort, with a crude HR of 0.81 (95% confidence interval [CI]: 0.64–1.02, *p* = 0.076). After adjusting for potential confounders, the adjusted HR reached statistical significance at 0.80 (95% CI: 0.63–1.01, *p* = 0.045). When we incorporated competing risk analysis, the protective effect became more pronounced, with an adjusted HR of 0.72 (95% CI: 0.57–0.91, *p* = 0.005). The cumulative incidence of hip fractures was consistently lower in alpha-blocker users throughout the follow-up period (Figure A1).

### 3.3. Subgroup Analysis

Subgroup analyses were performed to evaluate the risk of hip fracture among post-TURP patients with and without α-blocker use across different patient characteristics (Table 3). In the main model using 1:4 propensity score matching (*n* = 6853), α-blocker use showed a protective effect against hip fractures, with an adjusted hazard ratio (HR) of 0.80 (95% CI: 0.63–1.01, *p* = 0.045). Age-stratified analyses revealed varying effects across age groups: patients aged 50–65 years showed the strongest protective trend (adjusted HR: 0.56, 95% CI: 0.20–1.59), followed by those ≥80 years (adjusted HR: 0.83, 95% CI: 0.48–1.44) and 65–80 years (adjusted HR: 0.88, 95% CI: 0.62–1.25), although these differences did not reach statistical significance. Among comorbidity subgroups, patients with diabetes mellitus showed a significant protective effect from α-blocker use (adjusted HR: 0.43, 95% CI: 0.19–0.98, *p* = 0.042). Patients with hypertension demonstrated a stronger protective trend (adjusted HR: 0.75, 95% CI: 0.49–1.16) compared to those without hypertension (adjusted HR: 0.91, 95% CI: 0.63–1.33). In patients with and without dementia, the protective effect was maintained, although the small sample size in the dementia group resulted in wide confidence intervals (adjusted HR: 0.85, 95% CI: 0.10–7.23 for those with dementia; adjusted HR: 0.84, 95% CI: 0.63–1.12 for those without dementia) (Table 3).

## 4. Discussion

### 4.1. Summary of Key Findings

Our study demonstrates that continuation of selective alpha-blockers after TURP is associated with a reduced risk of hip fractures among older men with BPH. This finding is in contrast to those of previous studies that suggested an increased fracture risk with alpha-blocker use. The protective effect was particularly evident in our competing risk analysis, which showed an adjusted HR of 0.72 (95% CI: 0.57–0.91, *p* = 0.005), indicating a significant 28% reduction in hip fracture risk among long-term alpha-blocker users post-TURP. This effect may be because alpha-blockers are associated with reduced LUTSs, which are recognized as an independent risk factor for falls and fractures [21,22]. Our subgroup analyses also revealed several important patterns in the protective effect of alpha-blockers. The overall protective effect (adjusted HR: 0.80, 95% CI: 0.63–1.01, *p* = 0.045) was consistent across various patient subgroups, with particularly notable effects in specific populations. The most significant protection was observed in patients with diabetes mellitus (adjusted HR: 0.43, *p* = 0.042), suggesting a potentially synergistic effect in this high-risk population.

### 4.2. Mechanisms Underlying the Protective Effect

Although individuals who have undergone TURP are presumed to have no anatomic bladder outlet obstruction (BOO) [23], they still benefit from the continuation of alpha-blockers. This suggests that the therapeutic effects of alpha-blockers extend beyond simple BOO relief. The protective effects observed in this study can be explained by several inter-connected mechanisms as follows:

#### 4.2.1. The Impact of Alpha-Blockers on LUT Blood Perfusion

The multifactorial nature of LUTSs in patients with BPH involves both anatomical and functional components, particularly vasculature-related chronic ischemia. Pinggera et al. demonstrated significantly decreased lower urinary tract perfusion in elderly patients with LUTSs compared to asymptomatic controls [24]. A subsequent study showed that alpha-blocker administration was associated with a 132.8% increase in lower urinary tract perfusion, improved bladder capacity, and reduced international prostate symptom scores [25]. This improvement in tissue perfusion may have broader implications beyond urinary symptoms, potentially affecting the overall pelvic floor muscle function and stability.

#### 4.2.2. Adaptation to First-Dose Phenomenon and Long-Term Benefits of Alpha-Blockers

Although alpha-blockers initially cause hypotension-related adverse events through the first-dose phenomenon [26,27], this study’s findings suggest that this effect is temporary, as long-term users develop compensatory mechanisms for blood pressure stability. Alpha-blockers’ continued benefits after TURP extend beyond simple BOO relief by modulating autonomic nervous system activity [18], reducing nocturia episodes [28,29], and improving sleep quality [30]. This is particularly significant in patients with diabetes, who showed a more pronounced protective effect in this study [31]. Diabetes-related bladder dysfunction involves sympathetic overactivity that exacerbates storage symptoms through chronic hyperglycemia [32,33]. Alpha-blockers effectively target this sympathetic dysregulation, potentially explaining their enhanced protective effect in patients with diabetes by reducing nocturia-related fall risk while simultaneously improving peripheral insulin sensitivity and attenuating orthostatic hypotension [34].

#### 4.2.3. Role of Alpha-1 Adrenergic Receptors in Bladder Function Optimization and Its Anti-Inflammatory Effects

Alpha-1 adrenergic receptors are present not only in the prostate but also in the bladder neck, urethra, and pelvic floor muscles. Continued alpha-blocker therapy may optimize bladder-neck function, improve urethral compliance, enhance pelvic floor muscle coordination, and reduce overall LUT dysfunction [35]. These receptors may also exhibit anti-inflammatory properties in the LUT [36]. Chronic inflammation of the LUT is associated with increased tissue fibrosis, reduced blood flow, and impaired muscle function [37]. The anti-inflammatory effects of long-term alpha-blocker use may help maintain better tissue health and function.

Collectively, these factors contribute to improved physical function and a reduced risk of falls. This complex interplay of mechanisms suggests that the protective effect of alpha-blockers against hip fractures is multifactorial, and involves both direct physiological effects and indirect benefits through improved urinary function and quality of life. Understanding these mechanisms is crucial for optimizing treatment strategies in the post-TURP population, particularly in elderly patients at a higher risk of falls and fractures.

### 4.3. Comparison with Previous Studies and Methodological Considerations

Our findings contrast with those of several previous studies [7,8,9,10,11,12,13,14] that reported in-creased fracture risk with alpha-blocker use. This discrepancy can be attributed to several key methodological differences and unique aspects of our study design.

#### 4.3.1. The Follow-Up Duration Difference

Our study uniquely focused on long-term users who had been on alpha-blockers for at least 3 months before inclusion, whereas previous studies typically examined newly initiated users and observed outcomes within short timeframes (30–90 days) [11,12,13,14]. This distinction is crucial, as it potentially captures mainly adverse events associated with treatment initiation rather than long-term effects, particularly given the known first-dose phenomenon of alpha-blockers.

#### 4.3.2. The Study Patient Population Difference

The patient population in our study differed significantly from that in previous investigations [38]. Our cohort exclusively comprised post-TURP patients, representing a more homogeneous population with documented prostatic obstruction and subsequent surgical intervention. In contrast, earlier studies included heterogeneous populations of patients with BPH, many of whom may have had varying degrees of obstruction and different baseline risk factors. These selection criteria helped minimize confounding factors and provided more precise insights into the specific effects of alpha-blockers in a well-defined clinical context.

#### 4.3.3. The Analytical Approach Differences

We employed PSM and competing risk analysis, accounting for death as a competing event, which provides a more accurate estimation of hip fracture risk in the elderly population. Previous studies have primarily used traditional Cox proportional hazards models, which may overestimate the risk in populations in which competing events such as death are common [39]. Moreover, our statistical methodology was carefully adjusted for important confounders, such as diabetes, cardiovascular disease, and concurrent medications, which were not consistently addressed in earlier research.

#### 4.3.4. The Advancements in Alpha-Blocker Formulations and Clinical Practices

The timing of our study may have contributed to these different findings. Recent advances in alpha-blocker formulations and dosing strategies have led to better tolerability profiles compared to earlier generations of these medications. Our study period allowed for the assessment of modern alpha-blocker formulations, which may have better safety profiles than older agents previously studied [40]. Moreover, contemporary clinical practice tends to employ more careful patient selection and monitoring, particularly in the early phases of treatment, when the risk of orthostatic hypotension is highest. This evolution in clinical practice may explain why our modern cohort demonstrated better outcomes than those reported in historical studies [41].

#### 4.3.5. The Ethnic and Genetic Differences in Risk Assessment

Our study focused on the Asian population, particularly Taiwanese patients, and introduced important ethnic and genetic considerations. Previous studies have predominantly been conducted in Western populations, and genetic differences in drug metabolism, alpha-1 adrenergic receptor polymorphisms, and varying lifestyle factors may contribute to different risk profiles [7,8]. The lower body mass index typically observed in Asian populations, different dietary habits, and varying levels of physical activity may influence both the pharmacodynamics of alpha-blockers and the baseline risk of falls and fractures.

#### 4.3.6. Medication Adherence and Healthcare System Differences

We observed significant differences in medication adherence patterns. Our study specifically tracked long-term adherence through prescription records, whereas previous studies have often relied on intention-to-treat analyses or shorter follow-up periods. This methodological difference is particularly relevant, as the protective effects we observed may be partially attributed to better medication compliance and more consistent therapeutic levels. Furthermore, healthcare systems should also be considered when interpreting these contradictory results. Taiwan’s comprehensive National Health Insurance system provides regular access to healthcare services and medications, potentially leading to better monitoring and management of side effects. This systematic approach to healthcare delivery may contribute to better outcomes than studies conducted in healthcare systems with different access patterns or follow-up protocols.

#### 4.3.7. Subgroup Analysis and Potential Mechanisms

The pronounced protective effect in patients with diabetes (57% reduction in hip fracture risk) likely reflects multiple mechanisms. Recent research demonstrates that diabetic bladder dysfunction involves significant sympathetic overactivity with increased α1-adrenoceptor sensitivity, contributing to more severe nocturia even after TURP [42,43]. Each additional nocturnal void increases the fall risk by 28% in older adults with diabetes through disrupted sleep and orthostatic hypotension [44]. Alpha-blockers specifically reduce nocturia episodes in men with diabetes with BPH [45] while improving peripheral insulin sensitivity and attenuating orthostatic hypotension in diabetic autonomic neuropathy [46], potentially explaining the enhanced protective effect observed.

### 4.4. Clinical Implications and Future Directions

Our findings have several important clinical implications for post-TURP management.

#### 4.4.1. Guideline Reconsideration

The observed protective effect against hip fractures challenges conventional wisdom regarding alpha-blocker discontinuation after TURP. Current guidelines primarily focus on LUTS management and generally do not address long-term alpha-blocker use after TURP [47]. These guidelines may need revision to incorporate potential protective effects against hip fractures, particularly in high-risk populations.

#### 4.4.2. Individualized Treatment Approach

Rather than automatically discontinuing alpha-blockers after TURP, clinicians should consider individualizing treatment decisions based on patient-specific risk factors, including age, comorbidities, concurrent medications, and previous fall history [48]. This approach is particularly relevant for older patients with multiple comorbidities who face higher risks for both urinary symptoms and fractures.

#### 4.4.3. Cost-Effectiveness

The cost-effectiveness of continued alpha-blocker therapy is compelling. Using this study’s mean 3.8-year follow-up data, prolonged selective alpha-blocker use after TURP costs approximately USD 650 in the USA and USD 210 in Taiwan. In contrast, direct medical costs for hip fracture treatment often exceed USD 40,000 in the USA and USD 5000 in Taiwan [49]. Even preventing a modest proportion of these fractures could yield substantial economic benefits for both individuals and healthcare systems.

#### 4.4.4. Multidisciplinary Collaboration

This study’s findings have implications beyond urology, extending to primary care, geriatrics, and orthopedics. Improved coordination among these specialties could lead to more comprehensive care strategies addressing both urological symptoms and fall risk management [50,51]. This collaborative approach should include regular assessments of urinary symptoms and fall risk factors with appropriate medication adjustments. This expanded perspective represents a paradigm shift in alpha-blocker therapy for post-TURP patients—from solely treating urinary symptoms to serving as a broader preventive strategy for older adults’ health maintenance [52].

### 4.5. Limitations and Recommendations for Future Research

Despite the significant findings of this study, some limitations warrant consideration:

#### 4.5.1. Hip Fractures as a Surrogate for Fall Risk

This study used hip fractures requiring surgical intervention as the primary outcome measure rather than directly measuring fall incidence. While hip fractures in the older population are frequently associated with falls, they represent only one potential consequence of falls and are influenced by numerous factors beyond fall occurrence, including bone mineral density, frailty, comorbidities, and protective responses during falls. The relationship between falls and fractures is complex and non-linear, particularly in a population with varying degrees of frailty and comorbidities. Therefore, this study’s findings regarding reduced hip fracture risk with alpha-blocker use should not be directly interpreted as evidence of reduced fall incidence without further investigation.

#### 4.5.2. Study Design and Unmeasured Confounders

Despite using advanced statistical methods such as PSM and competing risk analysis, certain critical variables—including physical activity levels, dietary habits, smoking status, and body mass index—were unavailable in the database. These lifestyle factors can significantly influence both fall risk and fracture outcomes [53].

#### 4.5.3. Medication Exposure Assessment

This study’s reliance on prescription records to determine medication exposure presents inherent limitations. Our definition of continued alpha-blocker use (≥80% medication possession ratio with allowance for gaps ≤ 30 days) represents a balance between stringency and real-world prescription patterns. While this approach is consistent with established pharmacoepidemiologic methods, it was acknowledged that it may not capture all nuances of medication-taking behavior. While these records document medication dispensing, they cannot confirm actual consumption patterns. Without direct measures of adherence, such as pill counts or electronic monitoring devices, the actual exposure to alpha-blockers throughout the follow-up period cannot be verified. Future studies employing more granular measures of adherence and persistence would further refine our understanding of the dose–response relationship between alpha-blocker exposure and fracture risk.

#### 4.5.4. Sample Size in Subgroup Analyses

Some subgroup analyses, particularly the diabetic subgroup, resulted in wide confidence intervals despite showing substantial protective effects (adjusted HR: 0.43). These wide intervals indicate limited statistical power in specific subgroups, suggesting that the magnitude of the effect should be interpreted with caution.

#### 4.5.5. Generalizability

Since this study focused on the Taiwanese population, the findings may not be fully generalizable to other ethnic groups or healthcare systems. Several important regional differences warrant consideration: Alpha-blocker prescribing practices differ substantially between regions, with Asian healthcare systems favoring longer-term alpha-blocker monotherapy for BPH compared to Western countries’ preference for combination therapies. The specific agents prescribed also vary regionally, with silodosin and naftopidil more common in Asia versus tamsulosin in Western markets [54]. Hip fracture epidemiology shows notable variation, with Taiwan’s age-standardized incidence (392 per 100,000 person-years in men ≥ 65) falling between higher rates in Northern Europe and lower rates in Southern Europe [55]. These baseline differences may influence the absolute risk reduction achievable across populations. Patient characteristics differ significantly, with Asian populations generally having lower BMI, different bone mineral density patterns, and distinct dietary habits affecting bone health [56]. The prevalence of relevant comorbidities also varies regionally, with Taiwan’s diabetes rate (12.4%) exceeding the global average [57]. Finally, Taiwan’s universal healthcare coverage with low co-payments may result in better medication adherence and follow-up compared to systems with higher out-of-pocket costs, potentially affecting both treatment patterns and outcomes.

#### 4.5.6. Mechanism Clarification

The mechanism by which alpha-blockers may reduce hip fracture risk remains unclear. Although several potential pathways have been proposed, this study could not directly measure intermediate outcomes such as changes in urinary symptoms, sleep quality, or physical function.

Despite these limitations, our study provides strong evidence of the potential protective effects of long-term alpha-blocker use against hip fractures in post-TURP patients. The large sample size, extended follow-up period, and robust statistical methodology strengthen the reliability of the findings. Given the high prevalence of BPH and the significant morbidity associated with hip fractures in older adults, further investigation into this area remains an important public health priority.

## 5. Conclusions

This large-scale population-based cohort study provides compelling evidence that long-term alpha-blocker therapy in post-TURP patients is associated with a 28% reduction in hip fracture risk among the overall cohort and a 57% reduction among diabetic patients, challenging conventional perspectives on post-TURP medication management. Our findings, derived from comprehensive nationwide data and robust statistical analyses, suggest that the benefits of continued alpha-blocker therapy extend beyond traditional urinary symptom control and potentially include protective effects against serious fall-related injuries. The clinical implications are particularly relevant given the aging global population and the increasing prevalence of BPH requiring surgical intervention. The potential dual benefits of symptom control and fracture prevention suggest that the current paradigm of routine alpha-blocker discontinuation after TURP should be reconsidered, particularly in high-risk elderly patients. While this study’s findings demonstrate a significant association between alpha-blocker use and reduced hip fracture risk, it was emphasized that hip fractures serve as a surrogate endpoint and should not be directly equated with fall incidence, as fracture occurrence is influenced by multiple factors beyond falls, including bone quality, protective responses, and comorbidities.

Nonetheless, given the retrospective design, unmeasured factors such as physical activity, frailty, and lifestyle habits may have influenced fracture risk. While propensity score matching minimized confounding, residual biases remain possible. Future prospective studies are needed to confirm these findings and refine post-TURP management strategies.

## Figures and Tables

**Table 1:** Demographics.

Variables	1:4 PSM			
	Control	α-Blocker	Total	*p*-Value
N	5482	1371	6853	
Age	72.26 ± 7.58	72.24 ± 7.69	72.26 ± 7.60	0.906
Age group	-	-	-	0.669
50–65 y/o	913 (16.7%)	236 (17.2%)	1149 (16.8%)	
65–80 y/o	3609 (65.8%)	885 (64.6%)	4494 (65.6%)	
≥80 y/o	960 (17.5%)	250 (18.2%)	1210 (17.7%)	
HTN (%)	2596 (47.4%)	639 (46.6%)	3235 (47.2%)	0.620
DM (%)	1172 (21.4%)	292 (21.3%)	1464 (21.4%)	0.948
Hyperlipidemia (%)	826 (15.1%)	194 (14.2%)	1020 (14.9%)	0.393
CAD (%)	871 (15.9%)	212 (15.5%)	1083 (15.8%)	0.699
CVA (%)	691 (12.6%)	182 (13.3%)	873 (12.7%)	0.506
Chronic liver disease (%)	236 (4.3%)	64 (4.7%)	300 (4.4%)	0.557
Chronic renal failure (%)	86 (1.6%)	18 (1.3%)	104 (1.5%)	0.488
Osteoporosis (%)	82 (1.5%)	27 (2.0%)	109 (1.6%)	0.210
Dementia (%)	151 (2.8%)	42 (3.1%)	193 (2.8%)	0.536
Death (%)	1083 (19.8%)	288 (21.0%)	1371 (20.0%)	0.300
Hip fracture receiving surgery (%)	305 (5.6%)	57 (4.2%)	362 (5.3%)	0.037 *
Follow-up time (yr.)	3.91 ± 1.59	3.38 ± 1.74	3.80 ± 1.64	<0.001 *

Data are presented as n or mean ± standard deviation. * *p*-value < 0.05 was considered statistically significant after test. Abbreviations: HTN, hypertension; DM, diabetes mellitus; CAD, coronary artery disease; CVA, cerebral vascular accident.

**Table 2:** Analysis of the risk of hip fracture among the patients receiving surgery for BPH with and without α-blocker use.

Variables	1:4 PSM (*n* = 6853)	
	α-Blocker	
	Yes	No
Total	1371	5482
Hip fracture receiving surgery	83	541
Person-years	7137	39,622
Incidence rate ^a^	11.6	13.7
Univariate model		
crude HR (95% CI)	0.81 (0.64–1.02)	1 (ref.)
*p*-value	0.076	
Multivariate model ^b^		
aHR (95% CI)	0.80 (0.63–1.01)	1 (ref.)
*p*-value	0.045 *	
Competing risk ^b^		
aHR (95% CI)	0.72 (0.57–0.91)	1 (ref.)
*p*-value	0.005 *	

^a^ Per 1000 person-years. ^b^ Multivariate Cox proportional hazard regression model with adjustment for all baseline characteristics shown in Table 1. * *p*-value < 0.05 was considered statistically significant after test. Abbreviations: HR, hazard ratio; aHR, adjusted hazard ratio; CI, confidence interval; ref., reference.

**Table 3:** Subgroup analysis of the risk of hip fracture among the patients receiving surgery for BPH with and without α-blocker use.

Variables	1:4 PSM (*n* = 6853)			
	Crude HR ^a^ (95% CI)	*p*-Value	Adjusted HR ^a^ (95% CI)	*p*-Value
Main model				
No	1.00		1.00	
Yes	0.81 (0.64–1.02)	0.076	0.80 (0.63–1.01)	0.045 *
Age				
50–65 y/o				
No	1.00		1.00	
Yes	0.59 (0.21–1.67)	0.318	0.56 (0.20–1.59)	0.547
65–80 y/o				
No	1.00		1.00	
Yes	0.91 (0.64–1.29)	0.593	0.88 (0.62–1.25)	0.491
≥80 y/o				
No	1.00		1.00	
Yes	0.83 (0.48–1.44)	0.506	0.83 (0.48–1.44)	0.503
W/O HTN				
No	1.00		1.00	
Yes	0.94 (0.64–1.36)	0.732	0.91 (0.63–1.33)	0.634
W/I HTN				
No	1.00		1.00	
Yes	0.78 (0.50–1.20)	0.254	0.75 (0.49–1.16)	0.199
W/O DM				
No	1.00		1.00	
Yes	0.96 (0.71–1.30)	0.780	0.94 (0.69–1.27)	0.691
W/I DM				
No	1.00		1.00	
Yes	0.46 (0.20–1.07)	0.070	0.43 (0.19–0.98)	0.042 *
W/O Dementia				
No	1.00		1.00	
Yes	0.87 (0.65–1.15)	0.320	0.84 (0.63–1.12)	0.241
W/I Dementia				
No	1.00		1.00	
Yes	0.65 (0.08–5.29)	0.687	0.85 (0.10–7.23)	0.880

^a^ Cox’s proportional hazards model. * *p*-value < 0.05 was considered statistically significant after test. Abbreviations: HR, hazard ratio; CI, confidence interval; BPH: benign prostate hyperplasia; HTN, hypertension; DM, diabetes mellitus; W/O, without; W/I, with.

## Data Availability

All generated data are contained within this article.

## References

[1] Civinini R., Paoli T., Cianferotti L., Cartei A., Boccaccini A., Peris A., Brandi M.L., Rostagno C., Innocenti M. (2019). Functional outcomes and mortality in geriatric and fragility hip fractures-results of an integrated, multidisciplinary model experienced by the “Florence hip fracture unit”. Int. Orthop..

[2] Dubljanin-Raspopović E., Marković-Denić L., Marinković J., Nedeljković U., Bumbaširević M. (2013). Does early functional outcome predict 1-year mortality in elderly patients with hip fracture?. Clin. Orthop. Relat. Res..

[3] Verhamme K.M., Dieleman J.P., Bleumink G.S., van der Lei J., Sturkenboom M.C., Artibani W., Begaud B., Berges R., Borkowski A., Chappel C.R. (2002). Incidence and prevalence of lower urinary tract symptoms suggestive of benign prostatic hyperplasia in primary care—The Triumph project. Eur. Urol..

[4] Berry S.J., Coffey D.S., Walsh P.C., Ewing L.L. (1984). The development of human benign prostatic hyperplasia with age. J. Urol..

[5] Kok E.T., Schouten B.W., Bohnen A.M., Groeneveld F.P., Thomas S., Bosch J.L. (2009). Risk factors for lower urinary tract symptoms suggestive of benign prostatic hyperplasia in a community based population of healthy aging men: The Krimpen Study. J. Urol..

[6] Lerner L.B., McVary K.T., Barry M.J., Bixler B.R., Dahm P., Das A.K., Gandhi M.C., Kaplan S.A., Kohler T.S., Martin L. (2021). Management of Lower Urinary Tract Symptoms Attributed to Benign Prostatic Hyperplasia: AUA GUIDELINE PART I-Initial Work-up and Medical Management. J. Urol..

[7] Tae B.S., Jeon B.J., Choi H., Cheon J., Park J.Y., Bae J.H. (2019). α-Blocker and Risk of Dementia in Patients with Benign Prostatic Hyperplasia: A Nationwide Population Based Study Using the National Health Insurance Service Database. J. Urol..

[8] Cindolo L., Pirozzi L., Fanizza C., Romero M., Tubaro A., Autorino R., De Nunzio C., Schips L. (2015). Drug adherence and clinical outcomes for patients under pharmacological therapy for lower urinary tract symptoms related to benign prostatic hyperplasia: Population-based cohort study. Eur. Urol..

[9] Seo G.H., Lee Y.K., Ha Y.C. (2015). Risk of hip fractures in men with alpha-blockers: A nationwide study base on claim registry. J. Bone Metab..

[10] Jacobsen S.J., Cheetham T.C., Haque R., Shi J.M., Loo R.K. (2008). Association between 5-alpha reductase inhibition and risk of hip fracture. JAMA.

[11] Kahlaee H.R., Latt M.D., Schneider C.R. (2018). Association Between Chronic or Acute Use of Antihypertensive Class of Medications and Falls in Older Adults. A Systematic Review and Meta-Analysis. Am. J. Hypertens..

[12] Dave C.V., Li Y., Steinman M.A., Lee S.J., Liu X., Jing B., Graham L.A., Marcum Z.A., Fung K.Z., Odden M.C. (2024). Antihypertensive Medication and Fracture Risk in Older Veterans Health Administration Nursing Home Residents. JAMA Intern. Med..

[13] Welk B., McArthur E., Fraser L.A., Hayward J., Dixon S., Hwang Y.J., Ordon M. (2015). The risk of fall and fracture with the initiation of a prostate-selective α antagonist: A population based cohort study. BMJ.

[14] Seo J.H., Han J.S., Lee Y., Myong J.P., Ha U.S. (2022). Fall risk related to subtype-specific alpha-antagonists for benign prostatic hyperplasia: A nationwide Korean population-based cohort study. World J. Urol..

[15] Bird S.T., Delaney J.A., Brophy J.M., Etminan M., Skeldon S.C., Hartzema A.G. (2013). Tamsulosin treatment for benign prostatic hyperplasia and risk of severe hypotension in men aged 40-85 years in the United States: Risk window analyses using between and within patient methodology. BMJ.

[16] Chughtai B., Simma-Chiang V., Kaplan S.A. (2014). Evaluation and management of post-transurethral resection of the prostate lower urinary tract symptoms. Curr. Urol. Rep..

[17] Nitti V.W., Kim Y., Combs A.J. (1997). Voiding dysfunction following transurethral resection of the prostate: Symptoms and urodynamic findings. J. Urol..

[18] Kim S.J., Park S.G., Pak S., Lee Y.G., Cho S.T., Kwon O. (2022). Predictive factors for alpha blocker use after transurethral prostatectomy: Can preoperative urodynamic outcome predict alpha blocker medication after surgery?. PLoS ONE.

[19] Huang L.K., Chang Y.H., Shao I.H., Lee T.L., Hsieh M.L. (2019). Clinical Outcome of Immediate Transurethral Surgery for Benign Prostate Obstruction Patients with Acute Urinary Retention: More Radical Resection Resulted in Better Voiding Function. J. Clin. Med..

[20] Campbell J., Reid J., Ordon M., Welk B. (2019). The Utilization of Benign Prostatic Hyperplasia and Bladder-Related Medications After a Transurethral Prostatectomy. Urology.

[21] Parsons J.K., Mougey J., Lambert L., Wilt T.J., Fink H.A., Garzotto M., Barrett-Connor E., Marshall L.M. (2009). Lower urinary tract symptoms increase the risk of falls in older men. BJU Int..

[22] Liu P.S., Huang H.K., Ding D.C. (2021). Association of lower urinary tract symptoms and hip fracture in adults aged ≥50 years. PLoS ONE.

[23] Cooper K.L., McKiernan J.M., Kaplan S.A. (1999). Alpha-adrenoceptor antagonists in the treatment of benign prostatic hyperplasia. Drugs.

[24] Pinggera G.M., Mitterberger M., Steiner E., Pallwein L., Frauscher F., Aigner F., Bartsch G., Strasser H. (2008). Association of lower urinary tract symptoms and chronic ischaemia of the lower urinary tract in elderly women and men: Assessment using colour Doppler ultrasonography. BJU Int..

[25] Pinggera G.M., Mitterberger M., Pallwein L., Schuster A., Herwig R., Frauscher F., Bartsch G., Strasser H. (2008). alpha-Blockers improve chronic ischaemia of the lower urinary tract in patients with lower urinary tract symptoms. BJU Int..

[26] Chrischilles E., Rubenstein L., Chao J., Kreder K.J., Gilden D., Shah H. (2001). Initiation of nonselective alpha1-antagonist therapy and occurrence of hypotension-related adverse events among men with benign prostatic hyperplasia: A retrospective cohort study. Clin. Ther..

[27] Graham R.M., Thornell I.R., Gain J.M., Bagnoli C., Oates H.F., Stokes G.S. (1976). Prazosin: The first-dose phenomenon. Br. Med. J..

[28] Chang T.L., Kuo H.C. (2024). Nocturia, nocturnal polyuria, and nocturnal enuresis in adults: What we know and what we do not know. Tzu Chi Med. J..

[29] Pesonen J.S., Vernooij R.W.M., Cartwright R., Aoki Y., Agarwal A., Mangera A., Markland A.D., Tsui J.F., Santti H., Griebling T.L. (2020). The Impact of Nocturia on Falls and Fractures: A Systematic Review and Meta-Analysis. J. Urol..

[30] Silva J., Silva C.M., Cruz F. (2014). Current medical treatment of lower urinary tract symptoms/BPH: Do we have a standard?. Curr. Opin. Urol..

[31] Lin Y.H., Hou C.P., Chen T.H., Juang H.H., Chang P.L., Yang P.S., Lin Y.S., Chen C.L., Tsui K.H. (2017). Is diabetes mellitus associated with clinical outcomes in aging males treated with transurethral resection of prostate for bladder outlet obstruction: Implications from Taiwan Nationwide Population-Based Cohort Study. Clin. Interv. Aging.

[32] Fu Z., Wang F., Dang X., Zhou T. (2022). The association between diabetes and nocturia: A systematic review and meta-analysis. Front. Public Health.

[33] Yamaguchi C., Sakakibara R., Uchiyama T., Yamamoto T., Ito T., Liu Z., Awa Y., Yamamoto K., Nomura F., Yamanishi T. (2007). Overactive bladder in diabetes: A peripheral or central mechanism?. Neurourol. Urodyn..

[34] Moul S., McVary K.T. (2010). Lower urinary tract symptoms, obesity and the metabolic syndrome. Curr. Opin. Urol..

[35] Abreu-Mendes P., Silva J., Cruz F. (2020). Pharmacology of the lower urinary tract: Update on LUTS treatment. Ther. Adv. Urol..

[36] Nickel J.C., Touma N. (2012). α-Blockers for the Treatment of Chronic Prostatitis/Chronic Pelvic Pain Syndrome: An Update on Current Clinical Evidence. Rev. Urol..

[37] Black L.M., Lever J.M., Agarwal A. (2019). Renal Inflammation and Fibrosis: A Double-edged Sword. J. Histochem. Cytochem..

[38] Checcucci E., Veccia A., De Cillis S., Piramide F., Volpi G., Amparore D., Pecoraro A., Piana A., Granato S., Verri P. (2021). New Ultra-minimally Invasive Surgical Treatment for Benign Prostatic Hyperplasia: A Systematic Review and Analysis of Comparative Outcomes. Eur. Urol. Open Sci..

[39] Zhou J., Lee S., Liu X., Iltaf Satti D., Tai Loy Lee T., Hou In Chou O., Chang C., Roever L., Tak Wong W., Ka Chung Wai A. (2022). Hip fractures risks in edoxaban versus warfarin users: A propensity score-matched population-based cohort study with competing risk analyses. Bone.

[40] Yoosuf B.T., Panda A.K., Kt M.F., Bharti S.K., Devana S.K., Bansal D. (2024). Comparative efficacy and safety of alpha-blockers as monotherapy for benign prostatic hyperplasia: A systematic review and network meta-analysis. Sci. Rep..

[41] Haile E.S., Sotimehin A.E., Gill B.C. (2024). Medical management of benign prostatic hyperplasia. Clevel. Clin. J. Med..

[42] Chen S., Zhu Y., Feng X., Zhang Z., Li S., Shi B. (2014). Changes in alpha1-adrenoceptor and NGF/proNGF pathway: A possible mechanism in diabetic urethral dysfunction. Urol. Int..

[43] Jo J.K., Kim H., Bang W.J., Oh C.Y., Cho J.S., Shim M. (2023). Effect of Diabetes Mellitus on Symptomatic Improvement After Surgery for Benign Prostatic Hyperplasia in Patients With Lower Urinary Tract Symptom and its Relations With Prostatic Urethral Angulation. Int. Neurourol. J..

[44] Iinuma K., Nishino Y., Matsuoka K., Ihara T., Makabe S., Tanji R., Harigane Y., Ishida K., Tamaki M., Yokoi S. (2023). The prevalence and predictive factors of nocturnal polyuria in Japanese patients with nocturia: A multicentral retrospective cohort study. Sci. Rep..

[45] Stamatiou K., Lardas M., Kostakos E., Koutsonasios V., Michail E. (2009). The impact of diabetes type 2 in the pathogenesis of benign prostatic hyperplasia: A review. Adv. Urol..

[46] Caştur L., Uzunlulu M., Eken E., Çiçek M., Tahra A., Efiloğlu Ö., Demirtaş B., Yıldırım A. (2024). Effect of different alpha-receptor antagonists on metabolic parameters: A head-to-head comparison. Int. Urol. Nephrol..

[47] Hughes T., Harper P., Somani B.K. (2023). Treatment Algorithm for Management of Benign Prostatic Obstruction: An Overview of Current Techniques. Life.

[48] Ye Z., Wang J., Xiao Y., Luo J., Xu L., Chen Z. (2024). Global burden of benign prostatic hyperplasia in males aged 60-90 years from 1990 to 2019: Results from the global burden of disease study 2019. BMC Urol..

[49] Leytin V., Beaudoin F.L. (2011). Reducing hip fractures in the elderly. Clin. Interv. Aging.

[50] Lokeshwar S.D., Harper B.T., Webb E., Jordan A., Dykes T.A., Neal D.E., Terris M.K., Klaassen Z. (2019). Epidemiology and treatment modalities for the management of benign prostatic hyperplasia. Transl. Androl. Urol..

[51] Cameron I.D., Dyer S.M., Panagoda C.E., Murray G.R., Hill K.D., Cumming R.G., Kerse N. (2018). Interventions for preventing falls in older people in care facilities and hospitals. Cochrane Database Syst. Rev..

[52] Burgio K.L., Cunningham S.D., Newman D.K., Low L.K., Nodora J., Lipman T.H., Klusaritz H., James A.S., Rickey L., Gahagan S. (2023). Preferences for Public Health Messaging Related to Bladder Health in Adolescent and Adult Women. J. Womens Health.

[53] Seo G.H., Shim S.R., Lee H.W., Kim J.H., Chun D.I., Kim H.J., Lee H.Y., Kim J.H. (2018). Risk for Hip Fracture due to Alpha Blocker Treatment in Korean Women: National Health Insurance Database Study. Low. Urin. Tract. Symptoms.

[54] Chung S.D., Chang H.C., Chiu B., Liao C.H., Kuo H.C. (2011). The efficacy of additive tolterodine extended release for 1-year in older men with storage symptoms and clinical benign prostate hyperplasia. Neurourol. Urodyn..

[55] Masumori N., Tsukamoto T., Iwasawa A., Furuya R., Sonoda T., Mori M., Hokkaido Urological Disorders Conference Writing Group (2009). Ejaculatory disorders caused by alpha-1 blockers for patients with lower urinary tract symptoms suggestive of benign prostatic hyperplasia: Comparison of naftopidil and tamsulosin in a randomized multicenter study. Urol. Int..

[56] Kanis J.A., Odén A., McCloskey E.V., Johansson H., Wahl D.A., Cooper C., IOF Working Group on Epidemiology and Quality of Life (2012). A systematic review of hip fracture incidence and probability of fracture worldwide. Osteoporos. Int..

[57] Cheung C.L., Ang S.B., Chadha M., Chow E.S., Chung Y.S., Hew F.L., Jaisamrarn U., Ng H., Takeuchi Y., Wu C.H. (2018). An updated hip fracture projection in Asia: The Asian Federation of Osteoporosis Societies study. Osteoporos. Sarcopenia.

